# mInDel: a high-throughput and efficient pipeline for genome-wide InDel marker development

**DOI:** 10.1186/s12864-016-2614-5

**Published:** 2016-04-14

**Authors:** Yuanda Lv, Yuhe Liu, Han Zhao

**Affiliations:** Institute of Agricultural Biotechnology, Jiangsu Academy of Agricultural Sciences, Nanjing, 210014 China; Department of Crop Sciences, University of Illinois at Urbana-Champaign, Urbana, IL 61801 USA

**Keywords:** Next-generation sequencing (NGS), Insertions and deletions (InDels), Genome-wide marker discovery, Marker platform, Marker-assisted breeding

## Abstract

**Background:**

Rich in genetic information and cost-effective to genotype, the Insertion-Deletion (InDel) molecular marker system is an important tool for studies in genetics, genomics and for marker-assisted breeding. Advent of next-generation sequencing (NGS) revolutionized the speed and throughput of sequence data generation, and enabled genome-wide identification of insertion and deletion variation. However, current NGS-based InDel mining tools, such as Samtools, GATK and Atlas2, all rely on a reference genome for variant calling which hinders their application on unsequenced organisms and the output of short InDels compromised their use on gel-based genotyping platforms. To address these issues, an enhanced platform is needed to identify longer InDels and develop markers in absence of a reference genome.

**Results:**

Here we present mInDel (multiple InDel), a next-generation variant calling tool specifically designed for InDel marker discovery. By taking in raw sequence reads and assembling them into contigs *de novo*, this software identifies InDel polymorphisms using a sliding window alignment from assembled contigs, rendering a unique advantage when a reference genome is unavailable. By providing an option of combining multiple discovered InDels as output, mInDel is amiable to gel-based genotyping platforms where markers with large polymorphisms are preferred. We demonstrated the usability and performance of this software through a case study using a set of maize NGS data, and experimentally validated the accuracy of markers generated from mInDel.

**Conclusions:**

mInDel is a novel and practical tool that enables rapid genome-wide InDel marker discovery. The features of being independent from a reference genome and the flexibility with downstream genotyping platforms will allow a broad range of applications across genetics research and plant breeding. The mInDel pipeline is freely available at www.github.com/lyd0527/mInDel.

## Background

A robust system of molecular markers is instrumental to the quality and efficiency of various genetic and genomic research as well as marker-assisted breeding [1–3]. Compared to other markers, Insertion-Deletion (InDel) markers provide a number of advantages: InDels are an important and abundant form of genome variation, often more prevalent than other structural variants such as SSRs; InDels are potentially multi-allelic and co-dominant, offering more genomic information than the usually bi-allelic SNPs; Genotyping of InDel markers is technologically less demanding, whereas SNP detection usually requires expensive chemistries and equipment. The last advantage is especially appealing when a low-cost genotyping platform is preferred in any laboratory.

Recent advances of next-generation sequencing (NGS) technologies have significantly increased the speed and scale of sequence information generation, and greatly accelerated the discovery process for genetic markers [4]. The massive amount of data together with the short read nature of NGS nonetheless, created a hurdle for effective InDel variation mining. Even though software such as SAMtools [5], GATK [6] and Atlas2 [7] offer tools for variant discovery, their algorithms were not tailor for InDel discovery and therefore are limited to identifying short InDels (usually <10 bp). This prevented their use with traditional gel electrophoresis-based genotyping, where small polymorphisms are hard to score accurately. In addition, all current variant callers rely on the presence of a reference genome for read mapping, which significantly hampered their broad application on non-model organisms whose reference genomes are usually unavailable.

To address these issues, we developed the mInDel suite that is specialized in calling multiple contiguous InDels independent of a reference genome. Built upon *de novo* assembled scaffolds/contigs from short sequence reads, mInDel identifies multiple contiguous InDels and uses the combined cumulative variation for marker development. We confirmed the computing efficiency of our pipeline by testing on a set of maize sequencing data, and experimentally verified the high accuracy of the developed InDel markers. Flexible, easy to use and highly practical, this modular software takes in raw sequence reads and outputs InDel markers with PCR primer sequences designed. Together with its independence from a reference genome, mInDel is a useful resource to various genetic and genomic researches across species.

## Implementation

**Fig. 1:**
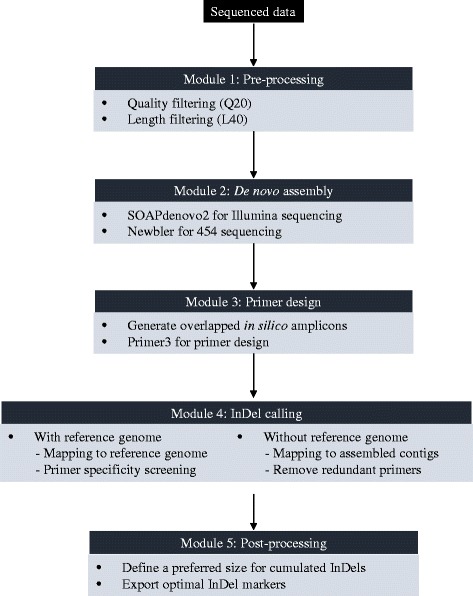
Overview of mInDel. mInDel is designed in five steps and implemented by five independent modules, which forms a pipeline of sequence analysis from raw sequences to InDel marker development. Modular design allows flexible choices of analysis steps based on users’ needs

In general, mInDel takes in raw sequence reads, pre-processes for quality control, and creates *de novo* assembled contigs. It then generates a pool of random primer pairs on the contigs and uses the inferred *in silico* amplicons to screen for InDel polymorphisms as candidate markers. Compared to direct polymorphism screening from contigs, the *in silico* amplicon design enables outputting developed InDel markers with PCR primer information ready, which further increases the practicality and efficiency of the marker development process. mInDel is implemented in five steps, with each step carried out by an independent module. The modularity allows users to run certain steps only based on their needs. A flow chart of the implementation is shown in Fig. 1.

### Module 1: Pre-processing analysis

Raw sequence files were taken in FASTQ format. Sequence coverage depths greater than 1x has been seen successfully run using mInDel. For quality control of sequence data, script *trim_fastq* takes in files in FASTQ format and filters against low quality, short reads and low complex regions by calling the SolexaQA++ program [8]. Single- and paired-end reads are both acceptable. In case that a sequence from a sequence-pair was removed, the remaining one is put into a separate file and used as a singlet during *de novo* assembly.

### Module 2: *De novo* assembly

The *de novo* assembly module provides two different assembly strategies for two commonly used platforms, Illumina (*de_novo_assembly_illu* script) and Sanger/454 (*de_novo_assembly_454* script). For the Illumina platform with relatively short reads, the SOAPdenovo2 [9] is adopted, which utilizes *de-Bruijn* graphs algorithm and accurately assembles sequences from short reads [10–12]. For Sanger or 454 platforms with relatively long reads, Newbler [13] incorporates the overlap algorithm to efficiently generating high quality assemblies from long reads. *De novo* assembly is necessary for samples that lack a reference genome.

### Module 3: Overlap primers design

This module generates a large pool of primer pairs, with inferred *in silico* amplicons in overlap spanning the entire input region. Overlapped amplicons are first generated using a sliding window method by *over_fragments* script, and then passed onto the Primer3 [14] program for primer design by *primers_design* script. The default window size is 300 bp, but can be readily changed to reflect users’ needs.

### Module 4: Paired-end mapping and specific screening

The mapping module is a module for inferring amplicon sizes between samples. The primer pool undergoes a paired-end mapping analysis using the Bowtie aligner [15, 16] with maximum insertion size < =1000 bp. Here, the value 1000 is set based on the consideration of single-pass PCR amplification ability. Users can increase the value for detecting longer InDels. Inferred amplicon sizes are then used to mine potential InDels among homologous loci between input entries. InDels with polymorphisms above the user-set threshold can be mapped to the reference genome (if available) for specific screening.

### Module 5: InDel screening and marker development

In the post-processing module, mInDel finalizes results and generates tab-delimited and Excel-compatible files by *InDel_screening* script. Accrued InDels facilitate genotyping from standard gel electrophoresis, but are also compatible with high-throughput fluorescent melting curve based genotyping assays. The output compiles information on optimum InDel markers including forward and reverse primers, product sizes, InDel sizes and chromosome locations ranked by a value score.

A set of test data is provided with mInDel for feature testing and performance check, which consists of simulated Illumina 100 bp*2 paired-end reads and raw reference sequences. Users are strongly encouraged to run this test with the command ‘*sh test.sh*’ after installation for runtime and performance check purposes, which usually takes about 5 minutes to run on a 8-core 2.5 GHz computer. The test data also serves as the foundation for example analyses described in the tutorial file.

## Results & Discussion

### Comparison with other InDel callers

We compared our mInDel pipeline with other InDel callers including SAMtools, GATK and Atlas2 (summarized in Table 1). By *de novo* assembling short reads into contigs, mInDel does not rely on a reference genome like other software do, which makes it feasible to identify InDel variants on samples with a partial or missing reference genome. mInDel also applies separate assembly algorithms for short and long sequencing reads, an approach that have been reported to help increase variant discovery accuracy [13]. Furthermore, mInDel can accumulate InDels into larger polymorphisms that are not only high-throughput genotyping compatible, but also PCR and gel electrophoresis friendly. This is especially appealing in scenarios where a low-cost genotyping approach is preferred.

**Table 1:** Comparison between mInDel with other InDel calling tools

Method	mInDel	SAMtools	GATK	Atlas2
*De novo* assembly	Yes	No	No	No
Reference genome-independent	Yes	No	No	No
Different aligners for different read lengths	Yes	No	No	No
InDel sized called	Short, Long	Short	Short	Short
High-throughput genotyping compatibility	Yes	Yes	Yes	Yes
PCR/electrophoresis genotyping amiability	High	Low	Low	Low

### InDel marker discovery in maize: a case study using mInDel

To demonstrate the functionality of mInDel, we obtained and analyzed a publicly available sequence data on four maize inbred lines. Due to its relatively large complex genome and the high percentage of repetitive sequences, maize would be a fitting organism for evaluating mInDel’s computational performance and processing proficiency. A total of 24 Gb, 36.9 Gb and 2.7 Gb of Illumina sequences from three maize inbred lines Mo17, Zheng58 and Qi319 were obtained from NCBI Sequence Read Archive (SRA) database. After pre-processing and *de novo* assembly, contigs for Mo17, Qi319 and Zheng58 were generated, which accounted for approximately 75 %, 19.5 % and 8.8 % of the B73 reference genome (Table 2).

**Table 2:** Summary of three genotypes genome data

Inbred Line	B73 genome	Mo17	Zheng58	Qi319
Raw data	/	24 Gb	36.9 Gb	2.7 Gb
Sequencing platform	/	Illumina	Illumina	Illumina
Cleaned data	/	21.2Gb	32.6Gb	2.1Gb
Assembled size	2.1G	1.5G	408.9 M	183.6 M
No. of Contigs	/	1,351,472	476,470	395,075
Longest contig	/	33,817 bp	18,068 bp	7,286 bp
N50 size	/	2,012 bp	1,157 bp	457 bp

We conducted two experiments as a comparison to show the distinctive feature of the InDel discovery independent of a reference genome sequence. Mo17 vs. B73 was an experiment to map Mo17 sequence reads to the maize B73 reference genome [17]; Zheng58 vs. Qi319 was the other experiment where Zheng58 sequence reads were mapped to the assembled Qi319 contigs, mimicking the scenario where a reference genome is unavailable. After primers design and paired-end mapping analysis, 4,205,672 primer pairs for Mo17 were generated and aligned against the B73 reference genome, and 610,091 Zheng58 primer pairs were generated and aligned against the *de novo* assembled Qi319 contigs. With a threshold of less than 3 mismatched bases, a total of 739,250 and 223,084 InDel loci from these two comparisons were successfully identified using mInDel (Table 3).

**Table 3:** Summary of InDel markers developed from two experiments

Types	Mo17 vs. B73 (with reference genome)	Zheng58 vs. Qi319 (without reference genome)
Total InDels	739,250	223,084
Mean size	71 bp	66 bp
1 ~ 10 bp	76,376	25,144
11 ~ 20 bp	63,714	21,291
21 ~ 100 bp	374,847	123,788
>100 bp	213,698	52,861
Discovery accuracy	86.8 % (276 out of 318)	72.6 % (437 out of 602)

The InDel discovery results from the aforementioned experiments demonstrated the unique feature of accrued polymorphism output 18.1 % and 19.9 % markers in these two experiments have an InDel of 1–20 bp, while 80.4 % and 80.1 % of markers have an InDel greater than 20 bp. The average length of the InDels was 71 bp and 66 bp, with the maximum InDels up to 387 bp and 371 bp respectively. Single-copied in silico amplicons with InDel size larger than 20 bp were selected and considered as optimum InDel markers for the ease of PCR detection and screening with agarose gel electrophoresis.

This pipeline was executed on a Dell PowerEdge 11G R910 (E7-4800 CPU) with 512 GB RAM under a Linux CentOS. The run time was 2 h 12 min for the Mo17 vs. B73 experiment with a fully assembled B73 genome, and 3 h 33 min for the Zheng58 vs. Qi319 experiment using assembled Qi319 contigs.

### Experimental validation of mInDel's discovery accuracy

To assess the accuracy of mInDel marker discovery, we conducted the following experimental validation. Primer pairs generated from mInDel were randomly sampled across the genome from the two aforementioned assigned groups and synthesized. PCRs were conducted in 20 μl reactions containing 10 μl HotStar Taq Master Mix (Qiagen, Germany), 1 μl of 10 μM primers, 2 μl DNA (50 ng/μl) and 7 μl water at 95 °C, 5 min; 34 cycles of (95 °C, 30 s; 58 °C, 30 s; 72 °C, 30 s) and 10 min extension at 72 °C. PCR products were electrophoresed on 2 % agarose gel and visualized with ethidium bromide. A high discovery accuracy rate was observed (Table 3), where 86.8 % (276 in 318 primer pairs) showed expected amplicon and polymorphism sizes from Mo17 vs. B73, and 72.6 % (437 in 602 primer pairs) in Zheng58 vs. Qi319.

### Computational Performance

mInDel is designed to allow processing NGS data on a standard desktop computer or a small server in a reasonable amount of time. Run time hinges on whether a reference genome is used to; for example, the run time was 2.5 hours the Mo17 vs. B73 experiment where B73 is a fully assembled genome, and 3.5 hours for the Qi319 vs. Zheng58 experiment where Zheng58 has assembled contigs. mInDel has been extensively tested using large datasets from various organisms. In general, Using 128 CPU threads with 2.00 GHz Intel Xeon E7-4850 Processor on a computational cluster, we were able to process 92 genomic libraries in ~11 hours, which demonstrated that mInDel is also well suited for population-scale analyses.

## Conclusions

To address the needs for a reliable and efficient InDel marker discovery tool, we developed this mInDel package that is able to output long InDel variants in absence of a fully assembled genome. This freely available and fully integrated suite offers demonstrated data processing performance, and provides great discovery accuracy. With its highly applied features, mInDel is a useful resource for various studies in genetics, genomics and marker-assisted breeding.

## Availability and requirements

Project home page: www.github.com/lyd0527/mInDel

Operating system(s): Linux, UNIX and OS X

Programming language: Perl and BASH

Other requirements: Bowtie, SOAPdenovo2 and Primer3.

License: GNU GPLv2

Any restrictions to use by non-academics: None

### Availability of data and materials

The data set supporting the results of this article is available in the NCBI SRA (Zheng58 from SRX120186; Qi319 from SRX121748; Mo17 from SRX026939).

## Abbreviations

NGS: next-generation sequencing
InDel: insertion and deletion
